# Conduction Mechanism and Improved Endurance in HfO_2_-Based RRAM with Nitridation Treatment

**DOI:** 10.1186/s11671-017-2330-3

**Published:** 2017-10-26

**Authors:** Fang-Yuan Yuan, Ning Deng, Chih-Cheng Shih, Yi-Ting Tseng, Ting-Chang Chang, Kuan-Chang Chang, Ming-Hui Wang, Wen-Chung Chen, Hao-Xuan Zheng, Huaqiang Wu, He Qian, Simon M. Sze

**Affiliations:** 1 Institute of Microelectronics, Tsinghua University, Beijing, 100084 China; 2 Tsinghua National Laboratory for Information Science and Technology (TNList), Beijing, 100084 China; 3 Department of Materials and Optoelectronic Science, National Sun Yat-Sen University, Kaohsiung, 80424 Taiwan; 4 Department of Physics, National Sun Yat-Sen University, Kaohsiung, 80424 Taiwan; 5 Advanced Optoelectronics Technology Center, National Cheng Kung University, Tainan, 70101 Taiwan; 6 School of Electronic and Computer Engineering, Peking University, Shenzhen, 518055 China; 7 Department of Electronics Engineering, National Chiao Tung University, Hsinchu, 300 Taiwan

**Keywords:** HfO_2_-based RRAM, Nitridation, Endurance, Space charge limit current

## Abstract

A nitridation treatment technology with a urea/ammonia complex nitrogen source improved resistive switching property in HfO_2_-based resistive random access memory (RRAM). The nitridation treatment produced a high performance and reliable device which results in superior endurance (more than 10^9^ cycles) and a self-compliance effect. Thus, the current conduction mechanism changed due to defect passivation by nitrogen atoms in the HfO_2_ thin film. At a high resistance state (HRS), it transferred to Schottky emission from Poole-Frenkel in HfO_2_-based RRAM. At low resistance state (LRS), the current conduction mechanism was space charge limited current (SCLC) after the nitridation treatment, which suggests that the nitrogen atoms form Hf–N–Ox vacancy clusters (V_o_^+^) which limit electron movement through the switching layer.

## Background

Recently, resistance random access memory (RRAM) composed of an insulating layer sandwiched by two electrodes has been widely studied as a promising candidate for next-generation nonvolatile memory due to its superior properties such as simple structure, low power consumption, high-speed operation (< 300 ps), and nondestructive readout [1–9]. Although most RRAM devices have many properties superior to nonvolatile memory, the high operation current of RRAM and performance degradation are major issues in nonvolatile memory in terms of the application of portable electronic products.

The Pt/HfO_2_/TiN structure can supply a conduction path which induces a resistive switching behavior [10–19]. However, the defects of amorphous HfO_2_ will increase the number of leakage paths, leading to power consumption and joule heating degradation. In this work, the resistive switching layer of HfO_2_ was treated by a solution with a urea/ammonia complex nitrogen source as the nitridation treatment to enhance its electrical switching properties.

## Methods

**Fig. 1:**
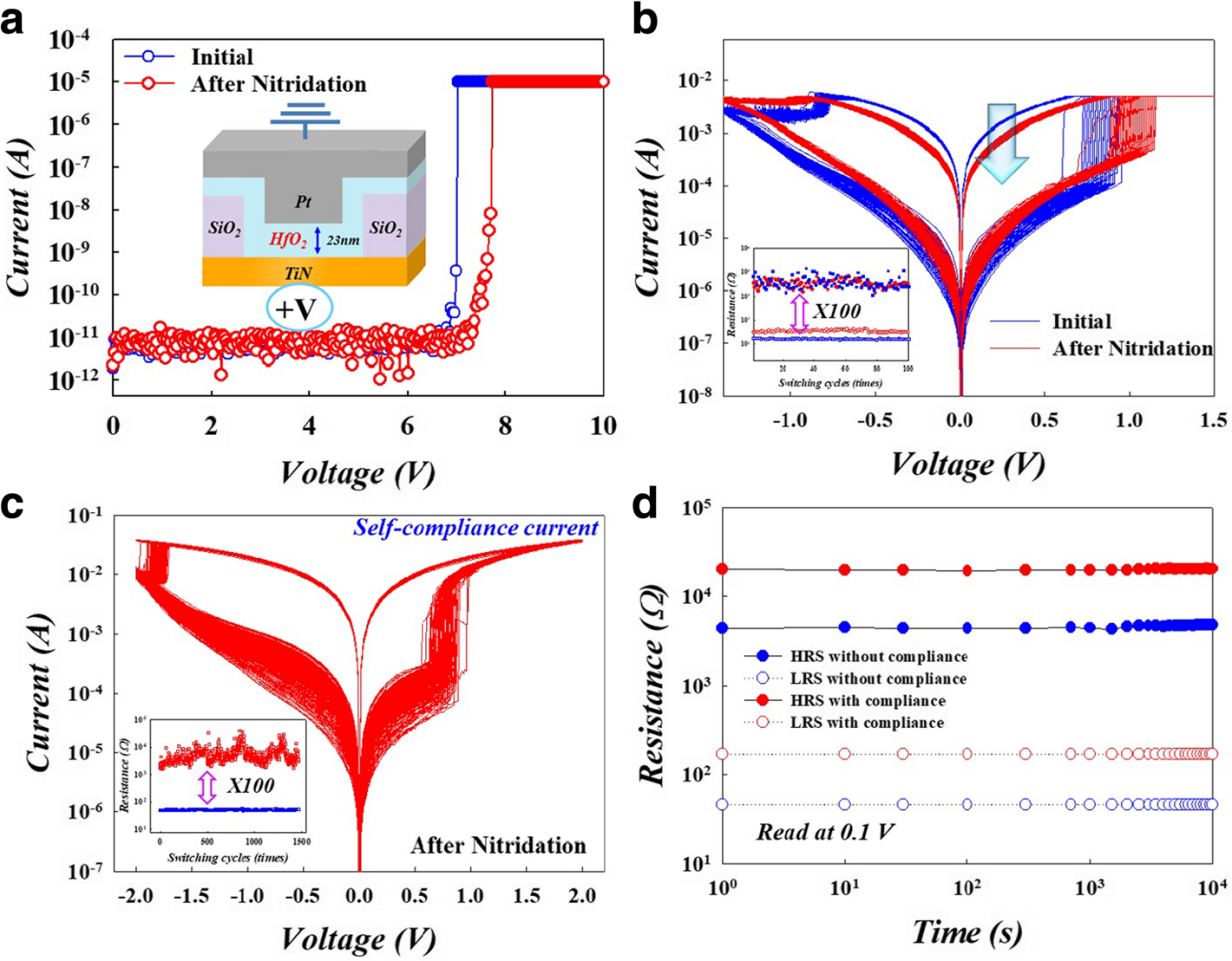
**a** The forming current curves of HfO_2_-based RRAM devices. **b** Comparison of DC sweep cycles at a 5 mA compliance current between initial and after nitridation treatment of HfO_2_-based RRAM. **c** DC sweep cycles without external current compliance of the HfO_2_ device after nitridation treatment. **d** Retention time of the HfO_2_-based RRAM devices at 85 °C with and without compliance current after nitridation treatment

The patterned TiN/Ti/SiO_2_/Si substrate was fabricated with a standard deposition and etching process, after which via holes can be formed (inset of Fig. 1a). Then, a 23-nm-thick HfO_2_ thin film was deposited into via holes on the substrate by RF magnetron sputtering using a pure HfO_2_ target. The sputtering power was fixed at RF power of 150 W and was carried out in argon ambient (Ar = 30 sccm) with a working pressure of 4 mtorr at room temperature. The HfO_2_/TiN semi-finished device was put into the reactive chamber and immersed into the solution with a urea/ammonia complex nitrogen source for nitridation treatment. During the nitridation treatment, the solution was heated to 160 °C in the system’s stainless steel chamber for 30 min. Then, the 110-nm-thick Pt top electrode was deposited by DC magnetron sputtering on the HfO_2_ thin film to form electrical devices with Pt/HfO_2_/TiN sandwich structures. Finally, all of the electric characteristics were measured by the Agilent B1500 semiconductor parameter analyzer. The DC and pulse sweeping bias were applied to the bottom electrode (TiN) while the top electrode (Pt) was grounded during the electrical measurements. In addition, Fourier-transform infrared spectroscopy (FTIR) was measured by a Bruker VERTEX 70v spectrometer in the middle infrared region.

## Results and Discussion

An electroforming process is required to activate all of the RRAM devices using a DC bias with a compliance current of 10 μA, as shown in Fig. 1a. After the forming process, the electrical current-voltage (I-V) properties of the HfO_2_-based RRAM were compared at initial and after the nitridation treatment. At LRS, the current was obviously reduced compared to that of untreated HfO_2_ thin film, as shown in Fig. 1b. The current reduction can be attributed to the defects passivated by the NH_3_ molecule in the treatment solution. We found that HRS distribution is much more stable after the nitridation treatment, as in the inset of Fig. 1b. The resistance states are extracted with a reading voltage of 0.1 V during the 100 sweep cycles with DC operation (inset of Fig. 1b). The resistance on/off ratio was slightly reduced after the nitridation treatment. Interestingly, a self-compliance resistive switching property was observed in these HfO_2_-based RRAM devices after the treatment, as shown in Fig. 1c. After more than 10^3^ sweep cycles, a repeatable self-protective characteristic of the device without hard breakdown was observed. The retention time was evaluated at 85 °C and remained stable even after 10^4^ s both in HRS and LRS.

**Fig. 2:**
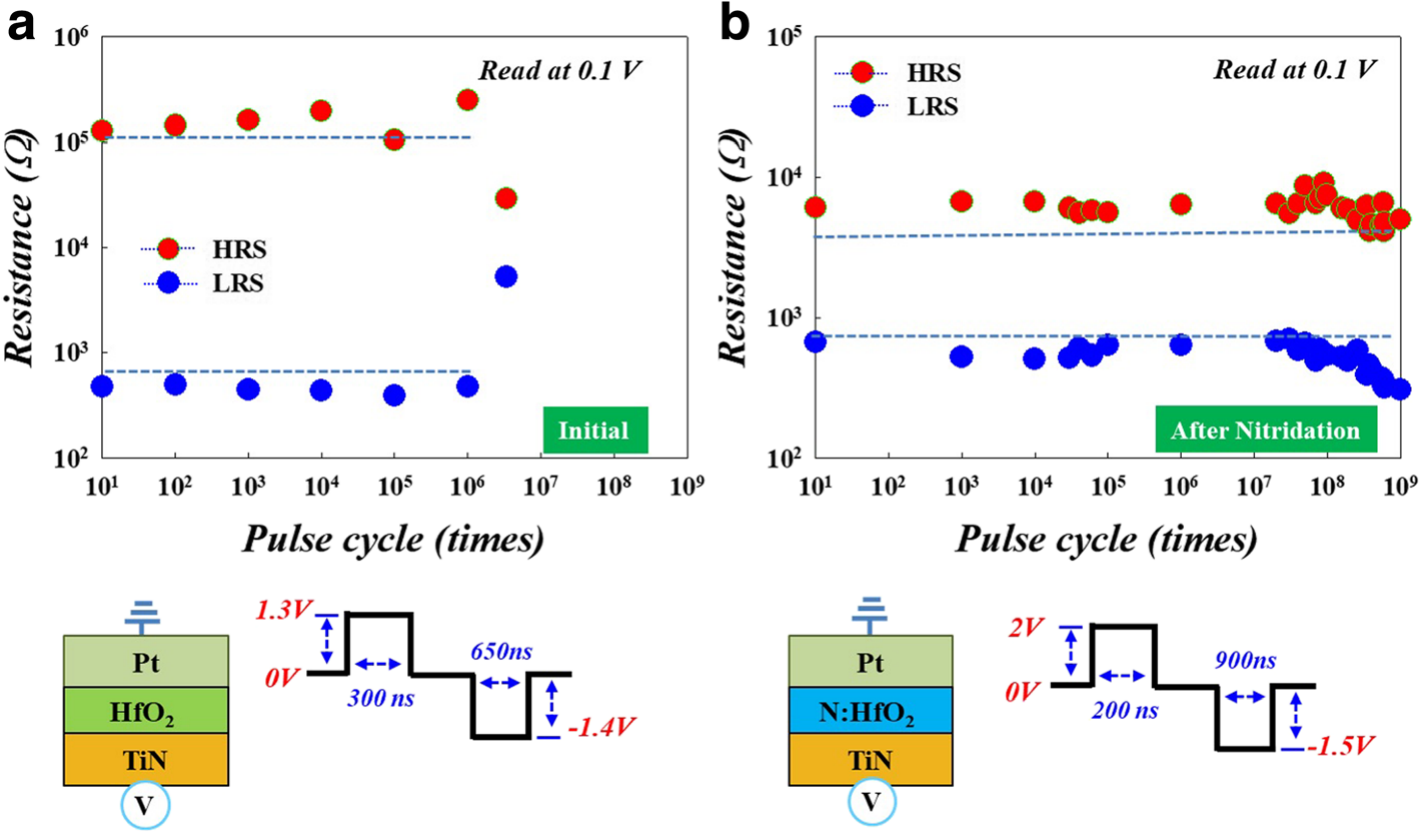
Comparison of endurance times in HfO_2_-based RRAM: **a** initial and **b** after nitridation treatment. The bottom diagrams are the corresponding device structures and endurance pulse conditions

**Fig. 3:**
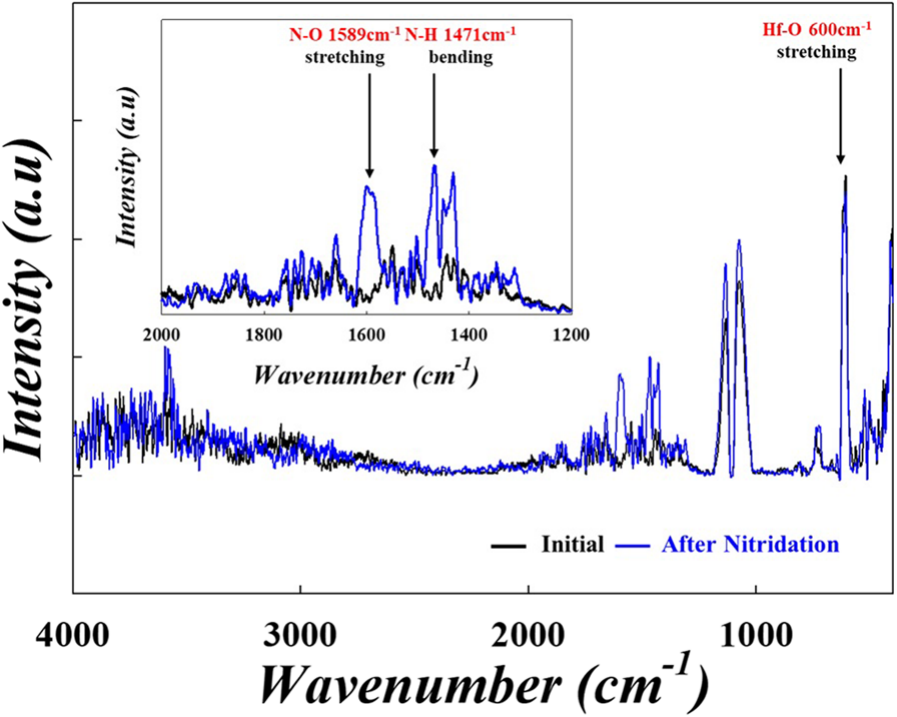
The comparison of FTIR spectra of HfO_2_ thin films between initial and after nitridation treatment

To further evaluate device performance, the endurance tests of HfO_2_-based RRAM were performed for initial and after the nitridation treatment, as shown in Fig. 2. In the untreated device after 10^6^ sweeping cycles, the HRS/LRS ratio significantly degrades from 100:1 to 5:1, as shown in Fig. 2a. After the nitridation treatment, however, even after more than 10^9^ sweep cycles, the device exhibited a stable HRS/LRS ratio, as in Fig. 2b. These results indicate that the nitridation process enhanced HfO_2_-based RRAM to perform with superior switching features and reliability. To further investigate these results, FTIR analysis was used to observe the chemical alterations of the HfO_2_ thin film, as shown in Fig. 3. A sharp peak at 1589 and 1311 cm^−1^ appeared after the nitridation treatment, corresponding to the symmetrical and asymmetrical stretching vibration peak of an N–O bond [20]. Further, the peak intensity of N–H bonds at 1471 cm^−1^ [21] increased due to the nitridation process by urea/ammonia complex nitrogen source (inset of Fig. 3). Therefore, we can infer the formation of nitrogen-containing compounds after the nitridation treatment.

**Fig. 4:**
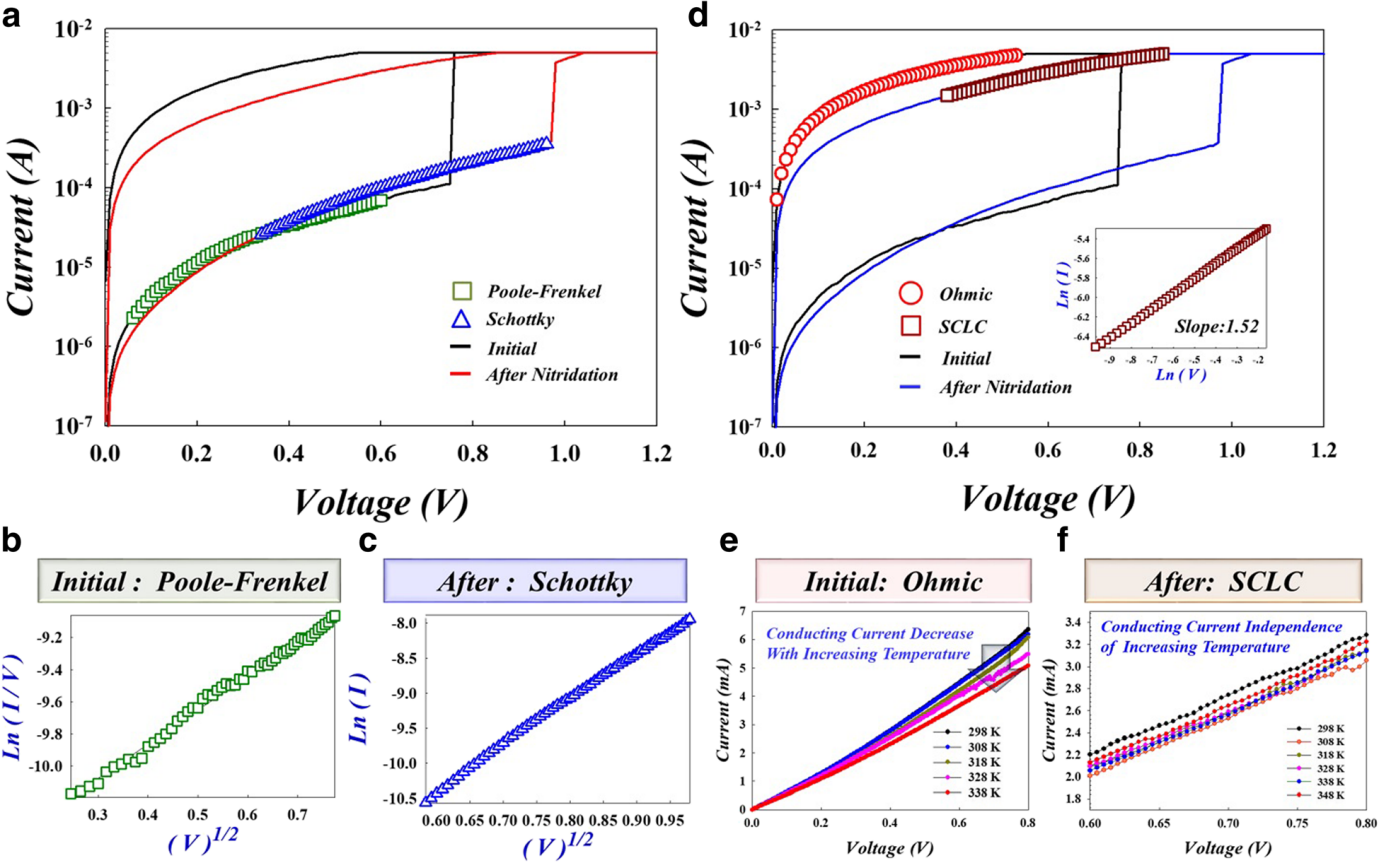
**a** Analysis current conduction mechanism of HRS from I-V curves in HfO_2_-based RRAM between initial and after nitridation treatment. **b** The Poole-Frenkel current conduction mechanism of HRS in HfO_2_-based RRAM. **c** The Schottky emission current conduction mechanism of HRS in HfO_2_-based RRAM after the nitridation treatment. **d** Analysis current conduction mechanism of LRS which transforms to SCLC from ohmic conduction after nitridation treatment in HfO_2_-based RRAM; the inset figure shows the SCLC current fitting result. **e** The Ohmic conduction mechanism of LRS in HfO_2_-based RRAM which is characteristic in current negative correlation with temperature. **f** The SCLC mechanism of LRS in HfO_2_-based RRAM that is independent on temperature after the nitridation treatment

In order to clarify the resistive switching mechanism, we analyzed the current conduction mechanism of the HfO_2_ thin film with and without the nitridation treatment, shown in Fig. 4a and d. For the untreated HfO_2_ thin film, the electrons were transferred through the defects, such that the current conduction mechanism was dominated by Poole-Frenkel conduction according to the linear relationship between ln(I/V) and the square root of the applied voltage (V^1/2^) on HRS, as shown in Fig. 4b [22].

In contrast, HfO_2_-based RRAM exhibited the Schottky emission mechanism according to the linear relationship between ln(I/T^2^) and the square root of the applied voltage (V^1/2^) of HRS, as shown in Fig. 4c [23, 24]. This is due to the decrease in defects and dangling bonds, as bonds become passivated by nitrogen atoms after the nitridation treatment. In addition, we also analyzed the current conduction mechanism with and without treatment at LRS in HfO_2_-based RRAM. On LRS, the carrier transport mechanism of the untreated HfO_2_-based RRAM was dominated by ohmic conduction, where current decreases with increasing temperature, as shown in Fig. 4e. After nitridation treatment, the current conduction mechanism transfers to space charge limited current (SCLC) with a slope of 1.52. The I-V curve is not relative to temperature, with a linear relationship between ln(I) and the square of the applied voltage V^2^ on LRS, as shown in Fig. 4f [25].

**Fig. 5:**
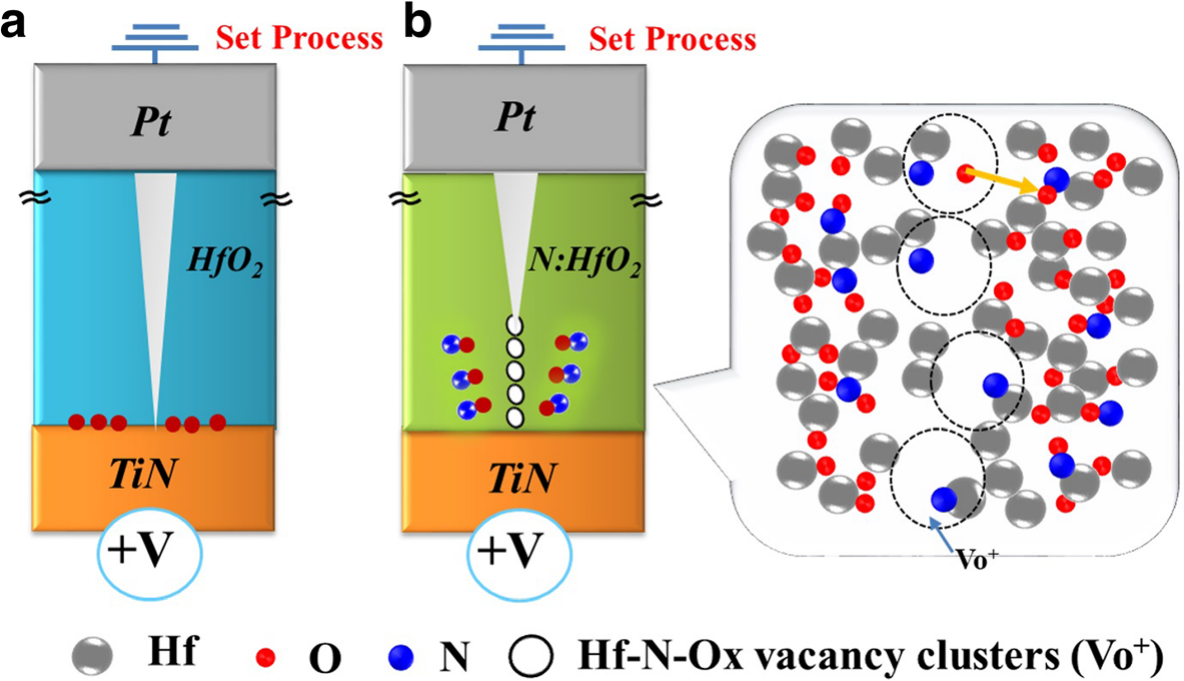
A schematic of the migration of oxygen ions through the set process in HfO_2_-based RRAM for **a** initial and **b** after nitridation treatment, which forms Hf–N–Ox vacancy clusters (V_o_^+^)

We proposed a model to explain the characteristics of the current conduction mechanism, and it is shown as Fig. 5. Thus, there are two offsetting dipoles associated with N and O atoms and a Hf atom (i.e., the sequence O–Hf–O is replaced by O–Hf–N–O) after doping N atoms into HfO_2_ thin film. Because nitrogen electron negativity is lower than oxygen, the dipole of Hf–N bond is lower than the Hf–O bond, which creates a lower dielectric constant region. When a positive bias is applied during the SET process, a series of Hf–N–Ox vacancies are formed due to their lower dielectric constant, then forming vacancy clusters (Vo^+^). The conductive path typically forms along with the Hf–N–Ox vacancy clusters (Vo^+^) as nitrogen atoms capture oxygen ions around the clusters, as shown in Fig. 5b. The presence of these insulating Hf–N–Ox vacancy clusters (Vo^+^) results in current reduction and the self-compliance effect found in HfO_2_-based RRAM.

## Conclusions

In summary, a self-compliance resistive switching property was observed in a Pt/HfO_2_/TiN RRAM device after the nitridation treatment. Endurance times reached 10^9^ cycles and a retention time of more than 10^4^ s was achieved at 85 °C. Due to the smaller electron negativity of the nitrogen atom when compared to the oxygen atom, the dipole of the Hf–N bond is smaller than that of the Hf–O bond, which creates a lower dielectric constant region. During the SET process, the Hf–N–Ox vacancy clusters (Vo^+^) form the conductive path. The insulating Hf–N–Ox vacancy clusters (Vo^+^) protect the device from hard breakdown and perform a self-compliance property.

## Abbreviations

FTIR: Fourier-transform infrared spectroscopy
HRS: High resistance state
LRS: Low resistance state
RRAM: Resistive random access memory
SCLC: Space charge limited current
